# Preoperative image-guided identification of response to neoadjuvant chemoradiotherapy in esophageal cancer (PRIDE): a multicenter observational study

**DOI:** 10.1186/s12885-018-4892-6

**Published:** 2018-10-20

**Authors:** A. S. Borggreve, S. Mook, M. Verheij, V. E. M. Mul, J. J. Bergman, A. Bartels-Rutten, L. C. ter Beek, R. G. H. Beets-Tan, R. J. Bennink, M. I. van Berge Henegouwen, L. A. A. Brosens, I. L. Defize, J. M. van Dieren, H. Dijkstra, R. van Hillegersberg, M. C. Hulshof, H. W. M. van Laarhoven, M. G. E. H. Lam, A. L. H. M. W. van Lier, C. T. Muijs, W. B. Nagengast, A. J. Nederveen, W. Noordzij, J. T. M. Plukker, P. S. N. van Rossum, J. P. Ruurda, J. W. van Sandick, B. L. A. M. Weusten, F. E. M. Voncken, D. Yakar, G. J. Meijer, on behalf of the PRIDE study group, B M P Aleman, R J H Borra, B van Etten, S S Gisbertz, L Goense, N Haj Mohammad, K J Hartemink, P Kappert, G Kats-Ugurlu, L L Kodach, T Korteweg, K K Krishnadath, J A Langendijk, W W J de Leng, S L Meijer, J H Potze, J Stoker, E Vegt, H M Verkooijen, S E Vollenbrock, F Wessels

**Affiliations:** 1 Department of Radiation Oncology, University Medical Center Utrecht, Utrecht University, Heidelberglaan 100, 3584 CX Utrecht, The Netherlands; 2 Department of Surgical Oncology, University Medical Center Utrecht, Utrecht University, Heidelberglaan 100, 3584 CX Utrecht, The Netherlands; 3 Department of Radiation Oncology, The Netherlands Cancer Institute - Antoni van Leeuwenhoek Hospital, Plesmanlaan 121, 1066 CX Amsterdam, The Netherlands; 4 Department of Radiation Oncology, University Medical Center Groningen, University of Groningen, Hanzeplein 1, 9713 GW Groningen, The Netherlands; 5 Department of Gastroenterology, Amsterdam University Medical Centers, Academic Medical Center, Meibergdreef 9, 1105 AZ Amsterdam, The Netherlands; 6 Department of Radiology, The Netherlands Cancer Institute - Antoni van Leeuwenhoek Hospital, Plesmanlaan 121, 1066 CX Amsterdam, The Netherlands; 7 Department of Radiology and Nuclear Medicine, Amsterdam University Medical Centers, Academic Medical Center, Meibergdreef 9, 1105 AZ Amsterdam, The Netherlands; 8 Department of Surgical Oncology, Cancer Center Amsterdam, Amsterdam University Medical Centers, Academic Medical Center, Meibergdreef 9, 1105 AZ Amsterdam, The Netherlands; 9 Department of Pathology, University Medical Center Utrecht, Utrecht University, Heidelberglaan 100, 3584 CX Utrecht, The Netherlands; 10 Department of Gastroenterology, The Netherlands Cancer Institute - Antoni van Leeuwenhoek Hospital, Plesmanlaan 121, 1066 CX Amsterdam, The Netherlands; 11 Department of Radiology, University Medical Center Groningen, University of Groningen, Hanzeplein 1, 9713 GW Groningen, The Netherlands; 12 Department of Radiation Oncology, Cancer Center Amsterdam, Amsterdam University Medical Centers, Academic Medical Center, Meibergdreef 9, 1105 AZ Amsterdam, The Netherlands; 13 Department of Medical Oncology, Cancer Center Amsterdam, Amsterdam University Medical Centers, Academic Medical Center, Meibergdreef 9, 1105 AZ Amsterdam, The Netherlands; 14 Department of Nuclear Medicine, University Medical Center Utrecht, Utrecht University, Heidelberglaan 100, 3584 CX Utrecht, The Netherlands; 15 Department of Gastroenterology and Hepatology, University Medical Center Groningen, University of Groningen, Hanzeplein 1, 9713 GW Groningen, The Netherlands; 16 Department of Nuclear Medicine, University Medical Center Groningen, University of Groningen, Hanzeplein 1, 9713 GW Groningen, The Netherlands; 17 Department of Surgical Oncology, University Medical Center Groningen, University of Groningen, Hanzeplein 1, 9713 GW Groningen, The Netherlands; 18 Department of Surgical Oncology, The Netherlands Cancer Institute - Antoni van Leeuwenhoek Hospital, Plesmanlaan 121, 1066 CX Amsterdam, The Netherlands; 19 Department of Gastroenterology, University Medical Center Utrecht, Utrecht University, Heidelberglaan 100, 3584 CX Utrecht, The Netherlands; 20 Department of Medical Oncology, University Medical Center Utrecht, Utrecht University, Heidelberglaan 100, 3584 CX Utrecht, The Netherlands; 21 Department of Pathology, University Medical Center Groningen, University of Groningen, Hanzeplein 1, 9713 GW Groningen, The Netherlands; 22 Department of Pathology, The Netherlands Cancer Institute - Antoni van Leeuwenhoek Hospital, Plesmanlaan 121, 1066 CX Amsterdam, The Netherlands; 23 Department of Pathology, Amsterdam University Medical Centers, Academic Medical Center, Meibergdreef 9, 1105 AZ Amsterdam, The Netherlands; 24 Department of Nuclear Medicine, The Netherlands Cancer Institute - Antoni van Leeuwenhoek Hospital, Plesmanlaan 121, 1066 CX Amsterdam, The Netherlands; 25 Imaging Division, University Medical Center Utrecht, Utrecht University, Heidelberglaan 100, 3584 CX Utrecht, The Netherlands; 26 Department of Radiology, University Medical Center Utrecht, Utrecht University, Heidelberglaan 100, 3584 CX Utrecht, The Netherlands

**Keywords:** Esophageal cancer, Neoadjuvant chemoradiotherapy, Pathologic complete response, Image-guided, MRI, DW-MRI, DCE-MRI, PET-CT, ctDNA

## Abstract

**Background:**

Nearly one third of patients undergoing neoadjuvant chemoradiotherapy (nCRT) for locally advanced esophageal cancer have a pathologic complete response (pCR) of the primary tumor upon histopathological evaluation of the resection specimen. The primary aim of this study is to develop a model that predicts the probability of pCR to nCRT in esophageal cancer, based on diffusion-weighted magnetic resonance imaging (DW-MRI), dynamic contrast-enhanced magnetic resonance imaging (DCE-MRI) and ^18^F-fluorodeoxyglucose positron emission tomography with computed tomography (^18^F-FDG PET-CT). Accurate response prediction could lead to a patient-tailored approach with omission of surgery in the future in case of predicted pCR or additional neoadjuvant treatment in case of non-pCR.

**Methods:**

The PRIDE study is a prospective, single arm, observational multicenter study designed to develop a multimodal prediction model for histopathological response to nCRT for esophageal cancer. A total of 200 patients with locally advanced esophageal cancer - of which at least 130 patients with adenocarcinoma and at least 61 patients with squamous cell carcinoma - scheduled to receive nCRT followed by esophagectomy will be included. The primary modalities to be incorporated in the prediction model are quantitative parameters derived from MRI and ^18^F-FDG PET-CT scans, which will be acquired at fixed intervals before, during and after nCRT. Secondary modalities include blood samples for analysis of the presence of circulating tumor DNA (ctDNA) at 3 time-points (before, during and after nCRT), and an endoscopy with (random) bite-on-bite biopsies of the primary tumor site and other suspected lesions in the esophagus as well as an endoscopic ultrasonography (EUS) with fine needle aspiration of suspected lymph nodes after finishing nCRT. The main study endpoint is the performance of the model for pCR prediction. Secondary endpoints include progression-free and overall survival.

**Discussion:**

If the multimodal PRIDE concept provides high predictive performance for pCR, the results of this study will play an important role in accurate identification of esophageal cancer patients with a pCR to nCRT. These patients might benefit from a patient-tailored approach with omission of surgery in the future. Vice versa, patients with non-pCR might benefit from additional neoadjuvant treatment, or ineffective therapy could be stopped.

**Trial registration:**

The article reports on a health care intervention on human participants and was prospectively registered on March 22, 2018 under ClinicalTrials.gov Identifier: NCT03474341.

## Background

Esophageal cancer is the ninth most common type of cancer and the sixth most leading cause of cancer related death [1]. Surgical resection has long been the standard curative treatment for locally advanced esophageal cancer. However, the poor survival rates of surgery alone prompted many researchers to explore neoadjuvant therapy approaches to improve survival. Randomized clinical trials have demonstrated a consistent prognostic benefit of neoadjuvant chemotherapy or chemoradiotherapy followed by surgery over surgery alone for locally advanced esophageal cancer [2–4]. In the Netherlands, this resulted in the adoption of neoadjuvant chemoradiotherapy (nCRT) according to the CROSS regimen followed by surgery as standard of care [4].

Nearly one third of all esophageal cancer patients (29%) treated with nCRT have no viable tumor cells detected at the primary tumor site at histopathological evaluation of the resection specimen, referred to as pathologic complete response (pCR) [4]. It has been argued that in patients who achieve a pCR, surgery may be omitted without substantially reducing survival outcomes. In fact, as an esophagectomy is associated with substantial morbidity, mortality (up to 3–5%) and impaired quality of life [5–9], it can be speculated that surgery may have a detrimental effect on these patients. Consequently, proper identification of pathologic complete responders prior to surgery could yield an organ-preserving regimen avoiding esophagectomy and its postoperative complications.

Reversely, 18% of patients have more than 50% vital residual tumor cells in the primary tumor bed at histopathology after nCRT and surgery, referred to as non-responders [4]. The CROSS regimen is associated with grade ≥ 3 toxicity events according to the Common Terminology Criteria for Adverse Events (CTCAE) in up to 13% of patients [4]. Thus, these non-responders are exposed to side effects of nCRT probably without the benefits. Therefore, early identification of the non-responders during nCRT may be beneficial, as alternative treatment strategies could be explored for this group, such as additional neoadjuvant treatment, or ineffective therapy could be stopped.

Several diagnostic strategies have been proposed to predict response and ultimately omit surgery in selected patients. Computed tomography (CT) is used preferably in initial staging of esophageal cancer, especially with regard to the presence of distant metastases, but does not satisfactorily restage after nCRT (accuracies ranging from 51 to 75%) [10–12]. Remaining tumor tissue is difficult to distinguish from therapy-induced peritumoral fibrosis and inflammation. As such, CT tends to overstage the preoperative tumor status.

Endoscopic ultrasonography (EUS) with or without biopsy has not yielded satisfactory results either. Systematic reviews pointed out that the accuracy rates of EUS for evaluating response to nCRT in esophageal cancer were moderate to poor (27–78%) [10, 12, 13]. The pooled sensitivity of EUS after nCRT for detection of residual primary tumor in a meta-analysis including 11 studies was 96.4% (95%-CI: 91.7–98.5%), with a pooled specificity of only 10.9% (95%-CI: 3.5–29.0%) [14]. Endoscopic biopsy after chemoradiotherapy for esophageal cancer on the other hand was a very specific (pooled estimate 91.0%, 95%-CI: 85.6–94.5%), but not a sensitive method (pooled estimate 34.5%, 95%-CI: 26.0–44.1%) for detection of residual primary tumor after nCRT, as reported in a recent meta-analysis including 12 studies [14]. Results from the recently published preSANO study revealed that with bite-on-bite biopsies, the sensitivity for the detection of residual disease increased substantially compared to regular biopsies (an increase from 54% [95%-CI: 38–70%] to 74% [95%-CI: 64–83%]) [15].

Moreover, promising results for response prediction were obtained using repeated integrated ^18^F-fluorodeoxyglucose positron emission tomography and computed tomography (^18^F-FDG PET-CT), with accuracies ranging from 76 to 85% [14, 16–18]. The change in ^18^F-FDG uptake during nCRT, reflecting a change in glucose metabolism by cancer cells, may be used to identify these responders [19]. A systematic review on the value of these quantitative ^18^F-FDG PET(-CT) measurements including 20 studies, showed that response could be predicted with sensitivities ranging from 33 to 100% (pooled estimate of 67%) and specificities ranging from 30 to 100% (pooled estimate of 68%) [19].

This supports the concept that functional imaging could play an important role in accurate response prediction. In this light, magnetic resonance imaging (MRI) has recently shown great potential for response prediction to nCRT for esophageal cancer [20–23]. Diffusion-weighted MRI (DW-MRI) is a functional imaging modality that allows for tissue characterization by deriving image contrast from restriction in the free diffusion (i.e. random mobility or Brownian motion) of water molecules, which is related to microstructural tissue organization. An apparent diffusion coefficient (ADC) map can be derived from the DW-MRI images to quantify the diffusion restriction in a certain volume of interest. The ADC is inversely correlated with tissue cellularity. As chemoradiotherapy can result in the loss of cell membrane integrity, tumor response can be detected as an increase in tumor ADC. In two exploratory studies, the treatment-induced relative changes in ADC over time (ΔADC), during nCRT, appeared highly predictive of histopathological response [22, 24]. Using repeated DW-MRI only, a high area under the receiver operating curve (AUC_ROC_) was attained for identifying pathologic complete responders was attained [22, 24].

Dynamic contrast-enhanced magnetic resonance imaging (DCE-MRI), the acquisition of serial MR images while intravenously administering a contrast agent, provides further insight into the nature of the tissue properties related to perfusion. Based on these images, quantitative parameters such as the transfer constant (K^trans^) and blood-normalized initial-area-under-the-gadolinium-concentration curve (AUC) can be calculated. The AUC reflects blood flow, vascular permeability and the fraction of interstitial space [16]. In a pilot study, all pathologic complete responders showed a decrease in AUC of 25% or more over the entire treatment course (ΔAUC), whereas an increase in AUC during treatment was observed for those patients who did not obtain a pCR (*p* = 0.003) [18].

In addition to functional imaging, circulating tumor cells and corresponding circulating tumor DNA (ctDNA) have been proposed as noninvasive and real-time biomarkers for predicting patient prognosis in esophageal carcinomas [25–28]. Circulating tumor cells and ctDNA are present in the blood vessels adjacent to the tumor, and are subsequently transported throughout the body via the circulation [27]. As such, ctDNA reflects the presence of disease and could provide valuable information on the response to treatment. Since ctDNA can be detected from regular peripheral blood samples, the detection of ctDNA could be a promising, minimally invasive addition to the evaluation of treatment response and prognosis in esophageal cancer patients.

### Study aim

As the aforementioned modalities do not individually fulfill the requirements to justify treatment decision making, the primary aim of the current study is to develop a multimodal prediction model that predicts the patients’ individual probability of a pCR after nCRT for esophageal cancer. Accurate prediction of the response to nCRT could lead to a patient-tailored approach with omission of surgery in the future in case of predicted pCR, potentially improving quality of life and reducing health care costs. Furthermore, additional neoadjuvant treatment could be offered to patients in case of non-pCR.

## Methods

### Objectives

The primary objective of the study is to develop a multimodal prediction model that predicts a patients’ individual probability of a pathologic complete response to nCRT in esophageal cancer by integrating DW-MRI, DCE-MRI and ^18^F-FDG PET-CT scans acquired prior to, during and after administration of nCRT.

The secondary objectives are as follows:

- To evaluate the accuracy of the multimodal prediction model as developed under the primary objective for the prediction of a pathologic good response (i.e. tumor regression grade [TRG] 1 or TRG 2).
- To evaluate the effectiveness and efficacy of an endoscopic and endosonographic assessment after nCRT for the detection of residual disease, in relation to the response classification as predicted by the model developed under the primary objective.
- To evaluate the presence of, and changes in, ctDNA during nCRT as a biomarker for a patients’ response to nCRT, the detection of residual disease after nCRT and progression-free and overall survival.
- To evaluate the accuracy of the multimodal prediction model as developed under the primary objective with addition of the endoscopic and endosonographic assessment, and the ctDNA measurements for the prediction of pCR and pathologic good response (i.e. TRG 1 or TRG 2).
- To evaluate the accuracy of a visual assessment for the detection of residual disease after nCRT based on MRI and ^18^F-FDG PET-CT.
- To evaluate the performance of MRI and ^18^F-FDG PET-CT imaging parameters for the prediction of progression-free and overall survival.

### Study design

The PRIDE study is a prospective, multi-center observational study with participation of 4 high-volume centers in the Netherlands (University Medical Center Utrecht, The Netherlands Cancer Institute - Antoni van Leeuwenhoek Hospital, University Medical Center Groningen and Amsterdam University Medical Centers). Patients will be informed and included at the outpatient department at one of these investigational centers. The study has been approved by the Medical Ethics Review Committee of the University Medical Center Utrecht (17–941, NL62881.041.17). All participating hospitals gave their consent after assessment of local feasibility. Written, voluntary, informed consent to participate in the study will be obtained from all patients.

### Study population

In order to be eligible to participate in this study, a patient must be scheduled to receive nCRT for a potentially resectable, locally advanced (cT1b-4aN0-3 M0) esophageal or gastroesophageal junction tumor, either squamous cell carcinoma or adenocarcinoma. Neoadjuvant chemoradiotherapy will be delivered according to the CROSS-regimen [4], consisting of weekly administration of carboplatin (doses titrated to achieve an area under the curve of 2 mg per milliliter per minute) and paclitaxel (50 mg per square meter of body-surface area) for 5 weeks and concurrent radiotherapy (41.4 Gy in 23 fractions, delivered 5 days per week on workdays with intensity modulated radiotherapy) followed by esophagectomy after 8–10 weeks. Primary diagnosis and staging will be based on endoscopy, EUS, ^18^F-FDG PET-CT and histopathological evaluation of a tumor biopsy.

Patients who meet exclusion criteria for MRI or for intravenous gadolinium-based contrast, patients with a blood plasma glucose concentration > 10 mmol/L or poorly controlled diabetes mellitus, patients with a status after endoscopic mucosal resection (EMR) or endoscopic submucosal dissection (ESD) of the primary tumor prior to the start of nCRT, patients younger than 18 years and pregnant or breast-feeding patients are not eligible.

### Study protocol

**Fig. 1:**
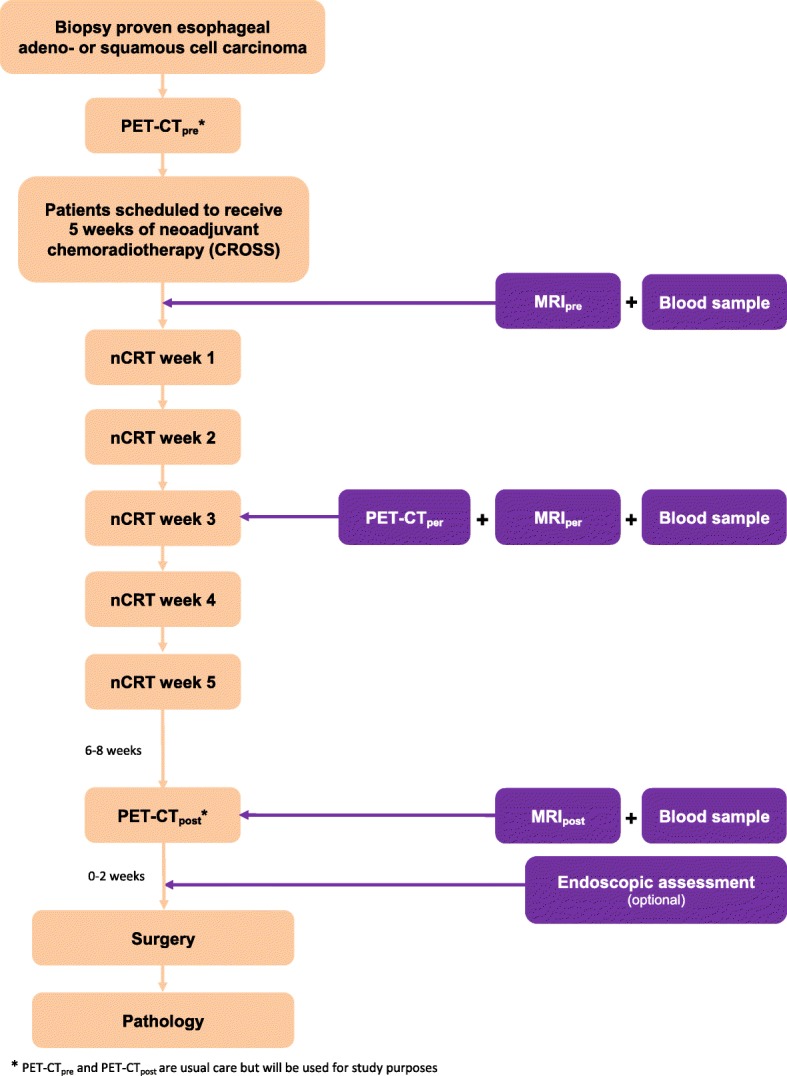
Study design

A schematic representation of the study protocol is depicted in Fig. 1. Patients will undergo standard diagnostic work-up and staging for esophageal cancer, including a baseline ^18^F-FDG PET-CT (PET-CT_pre_). After informed consent and before the start of nCRT, a baseline MRI (MRI_pre_) is performed. A second MRI (MRI_per_) and ^18^F-FDG PET-CT (PET-CT_per_) will be performed during the third week of nCRT (after 10–15 fractions of radiotherapy). A third MRI (MRI_post_) will be performed 6–8 weeks after the completion of nCRT, and no sooner than within 2 weeks before the intended date of surgery. The third ^18^F-FDG PET-CT (PET-CT_post_) is usual care in all participating centers, and will be performed within the same timeframe as the MRI_post_. Blood samples will be acquired at 3 time points, i.e. before, during and after nCRT, to evaluate the presence of, and changes in circulating tumor DNA (ctDNA). Furthermore, patients will be asked to undergo an additional endoscopic assessment after nCRT, PET-CT_post_ and MRI_post_, within 2 weeks prior to surgery. Surgical resection will be performed 8–10 weeks after completion of nCRT.

In summary, for study purposes patients will undergo 3 additional MRI scans (MRI_pre_, MRI_per_, MRI_post_), 1 additional ^18^F-FDG PET-CT scan (PET-CT_per_), blood samples at 3 time points and 1 postchemoradiation endoscopic and endosonographic assessment. The ^18^F-FDG PET-CT scans before start of nCRT and after nCRT (PET-CT_pre_ and PET-CT_post_) are standard of care in all participating centers and will also be used for study purposes. All study related procedures will take place before surgery.

#### MRI

Patients will undergo anatomical (T2-weighted [T2W]) and functional MRI (DWI and DCE) in one scanning session every time. Two DWI series and one DCE scan will be acquired. The sagittal DWI series (sagittal intravoxel incoherent motion [sIVIM] with 13 b-values: 0, 10, 20, 30, 40, 50, 75, 100, 200, 350, 500, 650 and 800 s/mm^2^) will be used for quantitative analyses but also for the visual assessment. Transversal DWI series (high resolution tDWI, b-values: 0 and 800 s/mm^2^)will mostly be used for visual assessments. The DCE-MRI scans will be acquired with a temporal resolution of 3 s and the injection of a gadolinium-based contrast agent. ADC and AUC values of the various time points will be used as quantitative measures of the DWI and DCE series, respectively. Extensive effort has been put in the standardization of MRI scan sequences by imaging experts and the exchange of test scans.

#### ^18^F-FDG PET-CT

The PET-CT examinations will be performed according to EARL guidelines (European Association of Nuclear Medicine) [29]. ^18^F-FDG is the tracer that will be used for the assessment of abnormal glucose metabolism in the tumor. On the ^18^F-FDG PET-CT scans, standardized uptake volumes (SUV_max_, SUV_mean_) and the total lesion glycolysis (TLG) will be measured to quantify changes over time in the glucose metabolism of the tumor.

#### Postchemoradiation endoscopic assessment

Patients will be asked to undergo a postchemoradiation endoscopic assessment, consisting of an additional endoscopy with (random) bite-on-bite biopsies of the primary tumor site and other suspected lesions in the esophagus, as well as endosonography with fine needle aspiration of suspected lymph nodes after completion of nCRT. This is an optional study procedure and patients can choose to opt-out for this additional procedure.

The endoscopic reevaluation will be performed by 1 or 2 experts in each of the 4 centers, to ensure high quality and uniform procedures and to reduce the impact of operator dependency. Furthermore, video recordings of all patients with negative biopsies that showed visual abnormalities of any kind during the endoscopic procedure will be reevaluated by an expert panel that will be blinded for the pathological outcome of the resection specimen in order to investigate whether a qualitative assessment of an expert team can help to correctly identify residual tumor in patients with a negative biopsy.

#### Blood samples

Blood samples will be used to evaluate the presence of ctDNA and changes in ctDNA concentrations since the release of ctDNA within a patient during the course of chemoradiotherapy has recently been demonstrated to be a dynamic process [30]. To allow molecular analysis of liquid biopsies, blood will be collected in cell-free DNA collection tubes. The plasma will be aliquoted after 2 centrifugation steps and will be stored at − 80 °C [31]. This will allow isolation of ctDNA and subsequent mutation analysis by means of Next Generation Sequencing (NGS) at a later stage.

#### Surgery

A transthoracic or transhiatal esophagectomy will be performed in all patients, depending on patient characteristics, tumor localization, and local preference. Open, hybrid and completely minimally invasive techniques are allowed. Resection of the primary tumor and regional lymph nodes will be carried out according to the current requirements for esophageal cancer surgery in the Netherlands [32]. For correct TNM-staging, the lymph node dissection should contain at least 15 nodes derived from both the mediastinum and upper abdomen.

#### Histopathological assessment

The resection specimen will be evaluated meticulously according to a standardized protocol (tumor type and extension, lymph nodes, resection margins) by a dedicated pathologist with gastrointestinal subspecialty in each center. The pathologist will be blinded for the results of the MRI and PET-CT exams. The most recent edition of the UICC (International Union Against Cancer) protocol will be used for TNM-classification and stage grouping [33]. Special attention will be given to reporting the effects of nCRT in the resection specimen. The (estimated) location of the primary lesion plus surrounding areas and other suspected lesions in the esophagus will be embedded in order to adequately judge the presence of residual tumor and treatment effects. The percentage of viable tumor cells will be scored microscopically (ranging from 0 to 100%), which directly corresponds to a stage in either of the two most often used grading systems: ‘TRG 1 to 4’ [34] or the ‘Mandard score 1 to 5’ [35]. Therapy effects include necrosis, inflammation with multinucleated giant cells, fibrosis and calcifications. Fibrosis is the most remarkable effect and is used to estimate the extension of the tumor before treatment. Lastly, all resection specimens with TRG 1–2 will be revised by a second expert pathologist.

#### Follow-up

Patients will remain in follow-up for 5 years after surgery, according to local follow-up policies. The general follow-up guideline in the Netherlands consists of routine follow-up visits every 3 months during the first year after surgery. In the second year, follow-up takes place every 6 months, and then yearly until 5 years after surgery. Diagnostic investigations are generally only performed on indication [32].

### Study outcomes

The primary outcome of this study is the performance of the multimodal prediction model for the correct prediction of a patients’ individual probability of a pCR to nCRT based on DW-MRI, DCE-MRI and ^18^F-FDG PET-CT scans acquired prior to, during and after administration of nCRT. Secondary outcome parameters include the performance of the model for good response (i.e. TRG 1 and TRG 2), the effectiveness and efficacy of a postchemoradiation endoscopic and endosonographic assessment for pCR prediction, the value of ctDNA as a biomarker for a patients’ response to nCRT, progression-free and overall survival, the performance of the model including results from the endoscopic and endosonographic assessment and ctDNA measurements for pCR and good response prediction, the performance of a visual assessment for the detection of pCR after nCRT based on MRI and ^18^F-FDG PET-CT, and lastly the performance of the model for prediction of progression-free and overall survival.

### Statistical analysis

#### Data analysis of primary study objective

The analysis regarding the primary objective of this project will have pCR as the predicted outcome of interest. Statistical analysis and reporting will be performed in accordance with the Standards for the Reporting of Diagnostic accuracy studies (STARD) statement, and the Transparent Reporting of a multivariable prediction model for Individual Prognosis or Diagnosis (TRIPOD) statement [36, 37]. The assessor(s) of the MRI and PET-CT images will be blinded for the histopathological outcome.

A multivariable logistic regression model will be developed with pCR as dichotomous outcome measure. Many of the imaging parameters are likely highly correlated and provide similar (non-additional) information, particularly within one modality. The most valuable imaging parameters within one imaging modality (i.e. within DW-MRI, DCE-MRI and PET-CT imaging parameters) will be entered in the model, based on the results of previous knowledge. To determine whether the imaging modalities provide complementary value in the prediction of pCR, models will be compared based on Akaike’s Information Criterion (AIC).

Model discrimination and calibration results will be evaluated for the multivariable logistic regression models using receiver operating characteristic (ROC) curve analysis with area-under-the-curve (AUC_ROC_) estimates and visual inspection of model calibration plots, respectively. Internal validation using the bootstrap method with 1000 repetitions will be carried out to provide insight into potential over-fitting and optimism in model performance. Bootstrapping will allow for calculation of bias-corrected c-indexes of the prediction model, and provides shrinkage factors that can be used to adjust the estimated regression coefficients in the model for overfitting and miscalibration. Sensitivity analyses will be performed excluding one participating center each time to study the influence of the multicenter study design on the model performance.

#### Data analysis of secondary study objectives

ROC curve analysis with AUC_ROC_ estimates will be used to determine the additional value of the postchemoradiation endoscopic and endosonographic assessment and the ctDNA measurements to the model as developed under the primary objective, as well as the accuracy of the multimodal prediction model for the prediction of pathologic good response (i.e. combined TRG 1 and TRG 2).

The performance of a visual assessment for the detection of pCR after nCRT will be analyzed by calculation of diagnostic performance measures such as sensitivity, specificity, positive predictive value, negative predictive value and accuracy (including corresponding 95% confidence intervals). This also applies to the individual performance of the endoscopic and endosonographic assessment for the detection residual disease, as well as for the performance of ctDNA measurements.

Multivariable Cox regression models will be used to analyze the performance of the prediction model as developed under the primary objective, MRI and ^18^F-FDG PET-CT imaging parameters, and ctDNA measurements for the prediction of progression-free and overall survival.

#### Sample size calculation

It is conservatively assumed that adenocarcinoma and squamous cell carcinoma need separate modeling and a priori stratification. Based on 3 independent imaging predictors (e.g. a DW-MRI imaging parameter such as ΔADC, a DCE-MRI imaging parameter such as ΔAUC and a ^18^F-FDG PET-CT imaging parameter such as ΔSUV_max_), this requires 30 events for both histopathological subtypes, according to the ‘1 predictor per ~10 events’ rule-of-thumb in logistic regression analysis [38, 39]. The CROSS-trial demonstrated a pCR rate of 23% and 49% after nCRT for patients with adenocarcinomas and squamous-cell carcinomas respectively [4]. According to the 1 in 10 rule, this translates in a total accrual of at least 130 adenocarcinoma patients and at least 61 squamous-cell carcinoma patients. In case of an unexpected aberrant distribution of patients that leads to decreased pCR rates, the aim is an accrual of 200 patients.

## Discussion

Currently, groups of patients with esophageal cancer fit in certain protocolled treatment approaches, but the treatment is rarely a perfect fit for the individual patient. The PRIDE study investigates whether a multimodal image-guided model can be developed that accurately predicts a patients’ individual histopathological response to nCRT. Such a model would enable personalized treatment for patients with esophageal cancer. Recent studies indicate that an organ-sparing approach might be feasible in selected patients with esophageal cancer who have a pCR after nCRT [40–42]. However, satisfactory diagnostic strategies to select these pathologic complete responders are lacking up to now. Therefore, surgical resection after nCRT remains the most optimal curative treatment in terms of survival in patients with locally advanced esophageal cancer. If the PRIDE concept provides high predictive performance for pCR, this could potentially lead to a new standard of care with direct benefits to esophageal cancer patients. Furthermore, accurate identification of the non-responders may be beneficial, as these patients might benefit from alternative treatment strategies, such as additional neoadjuvant treatment, or ineffective therapy could be stopped in this group.

In the current study protocol, strict time points are chosen for the MRI and ^18^F-FDG PET-CT imaging, as well as for the blood samples and endoscopic assessment. This way, a homogeneous cohort will be created, in which measurement variability will be reduced as much as possible. This is also reflected in the extensive effort of the participating centers to standardize the imaging protocols.

Since therapy effects continue to develop after treatment, previous studies have underlined that MRI and PET-CT imaging during nCRT as well as after nCRT for esophageal cancer can function as predictors for pCR [18, 43–46]. As such, the MRI_post_ and PET-CT_post_ scans should be as close to the histopathological assessment of the outcome (pCR) as possible, in order to make sure that the findings on the MRI_post_ and PET-CT_post_ will represent the histopathology accurately. This will likely also prevent false positive results caused by transient radiation-induced esophagitis, which is known to decrease over time after nCRT. Therefore, the chosen time points in our study include scans before the start of nCRT (MRI_pre_/PET-CT_pre_), scans during the third week of nCRT (MRI_per_/PET-CT_per_), and scans within 2 weeks before surgery (MRI_post_/PET-CT_post_). In patients undergoing an additional endoscopic and endosonographic assessment, this will be intended after the ^18^F-FDG PET-CT_post_ and the MRI_post_, but prior to surgery. If an organ-sparing approach is eventually implemented in clinical practice for predicted complete responders, an endoscopic confirmation without signs of residual tumor will most likely be required.

Of note, two studies, namely the Dutch SANO trial [47] and the French ESOSTRATE trial (ClinicalTrials.gov identifier NCT02551458), are currently studying active surveillance strategies after nCRT for patients with a clinical complete response. For the SANO trial, a clinical complete response is based on ^18^F-FDG PET-CT and endoscopy with at least 8 (random) bite-on-bite biopsies. These studies together will include a total of 600 patients (300 within each trial) and the primary outcome of these studies is survival. In contrast to these trials, the current study involves the careful development of an accurate image-guided response evaluation strategy to predict pCR in an observational study, without the simultaneous implementation of postponed surgical resection in clinical complete responders that might harm the patient. The results of this study will therefore play an important role in the accurate identification of esophageal cancer patients with a pCR to nCRT who could benefit from an organ-sparing approach in the future. Ultimately, the results of the three trials together could lead to a patient-tailored wait-and-see approach with omission of surgery in the appropriate patients.

## Abbreviations

^18^F-FDG PET-CT: ^18^F-fluorodeoxyglucose positron emission tomography and computed tomography
ADC: apparent diffusion coefficient
AIC: Akaike’s information criterion
AUC: area under the curve
CT: computed tomography
ctDNA: circulating tumor DNA
DCE-MRI: dynamic contrast-enhanced magnetic resonance imaging
DW-MRI: diffusion-weighted magnetic resonance imaging
EMR: endoscopic mucosal resection
ESD: endoscopic submucosal dissection
EUS: endoscopic ultrasonography
MRI: magnetic resonance imaging
nCRT: neoadjuvant chemoradiotherapy
NGS: next generation sequencing
PCA: principal component analysis
pCR: pathologic complete response
ROC: receiver operating curve
SUV: standardized uptake volume
T2W: T2-weighted
TLG: total lesion glycolysis
TRG: tumor regression grade
